# Phonon Transport in GaAs and InAs Twinning Superlattices

**DOI:** 10.1021/acs.jpcc.2c04859

**Published:** 2022-09-21

**Authors:** Kim López-Güell, Nicolas Forrer, Xavier Cartoixà, Ilaria Zardo, Riccardo Rurali

**Affiliations:** † Institut de Ciència de Materials de Barcelona, ICMAB−CSIC, Campus UAB, 08193 Bellaterra, Spain; ‡ Department of Physics, University of Basel, Klingelbergstrasse 82, 4056 Basel, Switzerland; § Departament d’Enginyeria Electrònica, Universitat Autònoma de Barcelona, Bellaterra, 08193 Barcelona, Spain; ⊥ Swiss Nanoscience Institute, University of Basel, Klingelbergstrasse 82, 4056 Basel, Switzerland

## Abstract

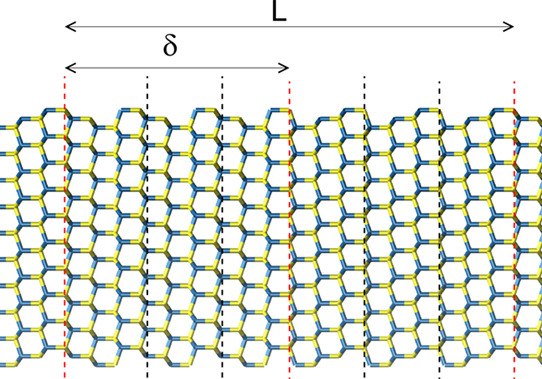

Crystal phase engineering gives access to new types of periodic nanostructures, such as the so-called twinning superlattices, where the motif of the superlattice is determined by a periodic rotation of the crystal. Here, by means of atomistic nonequilibrium molecular dynamics calculations, we study to what extent these periodic systems can be used to alter phonon transport in a controlled way, similar to what has been predicted and observed in conventional superlattices based on heterointerfaces. We focus on twinning superlattices in GaAs and InAs and highlight the existence of two different transport regimes: in one, each interface behaves like an independent scatterer; in the other, a segment with a sufficiently large number of closely spaced interfaces is seen by propagating phonons as a metamaterial with its own thermal properties. In this second scenario, we distinguish the case where the phonon mean free path is smaller or larger than the superlattice segment, pointing out a different dependence of the thermal resistance with the number of interfaces.

## Introduction

The design of materials with tailor-made thermal properties is very attractive for several applications, ranging from efficient thermoelectrics^1,2^ to thermal management.^3^ A way to engineer the phonon spectrum of a material, and thus to tune its thermal conductivity, is by creating superlattices, where wave interference creates forbidden energy band gaps for phonons.^4,5^ An additional interest in superlattices is that they allow, in principle, to observe the crossover from a particle- to a wave-like phonon transport regime, a topic of both fundamental and applied importance. When phonons travel across far-apart interfaces, they are better described as particles that suffer multiple independent diffusive scattering events, each one characterized by the thermal boundary resistance (TBR) of that interface.^6−8^ When the number of interfaces or their density increases, interference effects can build up, and heat transport is better understood by taking into account the wave nature of phonons. In the first situation, the thermal conductance is tuned by controlling the number of interfaces; once the coherent regime kicks-in, on the other hand, the main control knob becomes the periodicity of the superlattice, which, in turn, determines the details of the phonon dispersion of the metamaterial, including the position and width of the phonon band gaps.^5,9−11^ This transition typically occurs by making the interface spacing of the same order of the phonon mean free path,^9^ a goal that can be achieved by either increasing the interface density (i.e., reducing the superlattice period) or decreasing the temperature.^12,13^ Experimental indications of these effects have been reported in GaAs/AlAs^14^ and perovskite oxides superlattices.^15^ In this scenario, the quality of each individual interface is almost as important as their periodic arrangement.^16,17^ Indeed, atomic-scale corrugations^16,18−21^ and interfacial chemical mixing^22,23^ have been shown to largely suppress the coherence of phonon transport. Therefore, the design of unconventional periodic structures, beyond the usual heterostructured superlattices, is attracting considerable attention.^24−26^

**Figure 1:**
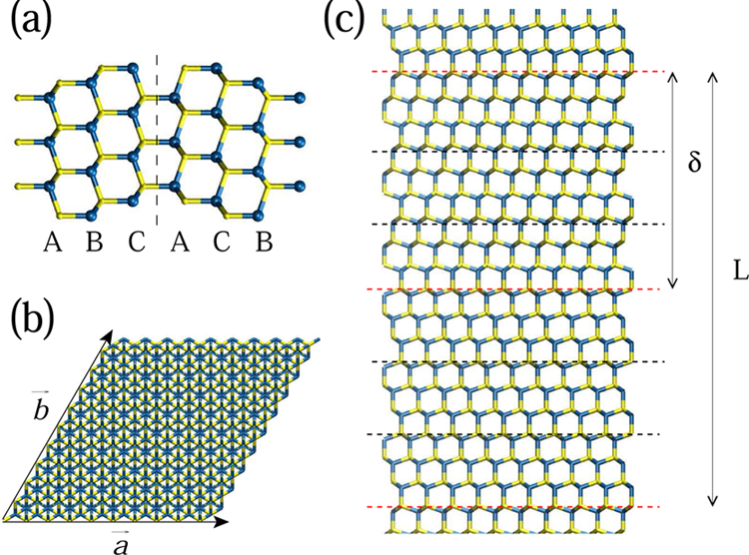
(a) Zoomed view of a twin defect, showing the change from ABC to ACB stacking. (b) Cross-sectional view of the computational cell, where *a* = *b* = 39.97 Å for GaAs and 42.85 Å for InAs. (c) Segment of a TSL containing one full period, *L* = 2δ, where δ is the separation between adjacent twins. The red dashed lines indicate the position of the twins, while the black dashed line shows the unit cell of the zincblende, when the [111] crystal axis is taken to be parallel to the cartesian *z*-axis. Blue spheres represent Ga or In atoms, and yellow spheres represent As atoms.

The increased control in the growth of semiconducting nanowires (NWs)^27,28^ has brought attention to a novel type of superlattice, where, rather than chemically different materials, different crystal phases of the same material—typically zincblende and wurtzite segments of arsenides and phosphides—alternate with a well-defined periodicity. These crystal phase superlattices^29−31^ are made of homojunctions that have (i) minimal lattice mismatch and (ii) no chemical intermixing and thus are virtually atomically flat. An even subtler kind of periodic superstructure that can be obtained in NWs is the so-called twinning superlattice (TSL).^32,33^ Here, both the material and the crystal phase are the same throughout the entire NW length, and the motif of the superlattice is determined by a periodic rotation of the crystal lattice, which induces the formation of a stacking planar defect that we refer to as a twin boundary. TSLs of practical interest are made of zincblende materials that feature a 60° rotation of the crystal lattice, so that the ABC stacking along the [111] crystal axis switches to ACB after each twin boundary (see the sketch in Figure 1a); see ref (34) for other types of twin boundaries.

Twin boundaries are peculiar interfaces under many respects and defy the most common phenomenological approaches to the calculation of the TBR, that is, the thermal resistance of an interface.^6−8^ The popular acoustic mismatch model (AMM) computes the TBR in terms of the mismatch of elastic properties of the constituent materials forming an interface. The rationale behind it is that a phonon impinging on the interface from one side is efficiently transmitted only if a suitable vibrational state, in terms of energy and momentum, exists on the other side. In a twin boundary, however, the materials on the two sides of the interface are identical and the AMM predicts no TBR, at odds with experimental results that have convincingly shown that they do have an effect on phonon dispersion^35^ and with theoretical atomistic simulations of phonon transport.^25,36^ The diffuse mismatch model (DMM), on the other hand, returns the same finite value of the TBR that it would erroneously attribute to a homogeneous system without interface; this is a known shortcoming of the DMM. If one considers a homogeneous system, without any physical interface, and applies the recipe of the DMM across a fictitious boundary, a finite TBR is obtained. Previous theoretical calculations, combining molecular dynamics and Green’s functions, showed that the TBR of twin boundaries in GaP and InP is determined by the rotation of the phonon polarization vectors and by local structural distortion at the interface.^36^ This explains why simplified models based on the mismatch of the elastic properties cannot capture phonon scattering at twin boundaries and call for modeling approaches that explicitly account for the atomic structure of the interface. Other systems falling in this category include some kind of domain walls in ferroelectrics^37,38^ or inversional interfaces in two-dimensional Janus monolayers, similar to those discussed in a recent report.^39^

## Computational Methods

We perform non-equilibrium molecular dynamics (NEMD) simulations with the LAMMPS code^40^ and a Tersoff-type interatomic potential^41^ parameterized by Nordlund and co-workers.^42^ We consider GaAs and InAs computational cells with the transport direction parallel to the cubic [111] crystal axis, which we take to be the *z* coordinate direction. This choice is dictated by the fact that this is the direction along which twin boundaries can be formed during NW growth. The ends of the computational cell are connected to Nosé–Hoover thermostats at temperatures *T*_H_ and *T*_C_, while the rest of the system evolves according to the microcanonical ensemble. We start from the 6-atom unit cell of zincblende crystals, which has the [111] crystallographic direction parallel to the *z*-axis, and construct 10 × 10 × *M* supercells. We take *M* = 90 for the study of isolated interfaces and *M* = 180 for systems featuring multiple interfaces, that is, a superlattice segment. We apply periodic boundary conditions along the *x*- and *y*-axes. The TBR is calculated as Δ*T*/*J*, where Δ*T* is the temperature jump at the interface and *J* is the heat flux^6^ (see refs (7), (43), and (44) for a more general discussion).

After the simulation starts, a thermal gradient rapidly builds up, but we nevertheless disregard the first 3 × 10^6^ steps to allow a proper equilibration of the system. In all cases, this time interval proved to be sufficient to reach the nonequilibrium steady state. Indeed, after this equilibration interval, not only the time evolution of the local temperature along *z* is roughly constant but also the rate of energy injected and extracted by the hot and cold thermostats is the same, within numerical fluctuations. After the steady state is reached, we average the heat flux and the temperature overruns that go from 7.5 to 30 × 10^6^ steps. We apply a temperature difference *T*_H_ – *T*_C_ equal to 100 K, varying *T*_C_ and *T*_H_ in order to obtain a different average temperature, *T*_M_. Notice that below the Debye temperature, the use of classical dynamics, where quantum effects are neglected, should be handled with care. Yet, attempts at correcting for quantum features in low-temperature molecular dynamics provided inconclusive and contrasting results.^45^ We address the effect of this limitation in detail below.

NEMD notoriously suffers from finite-size effects, and the usual procedure to estimate the thermal conductivity of a material requires running simulations in increasingly large cells. In this work, however, our goal is either computing TBRs, which is much less sensitive to cell sizes,^46^ or studying the dependence of the thermal resistance in multi-interface systems, where we compare results obtained in cells of the same size.

## Results and Discussion

### Isolated Twin Boundary

**Figure 2:**
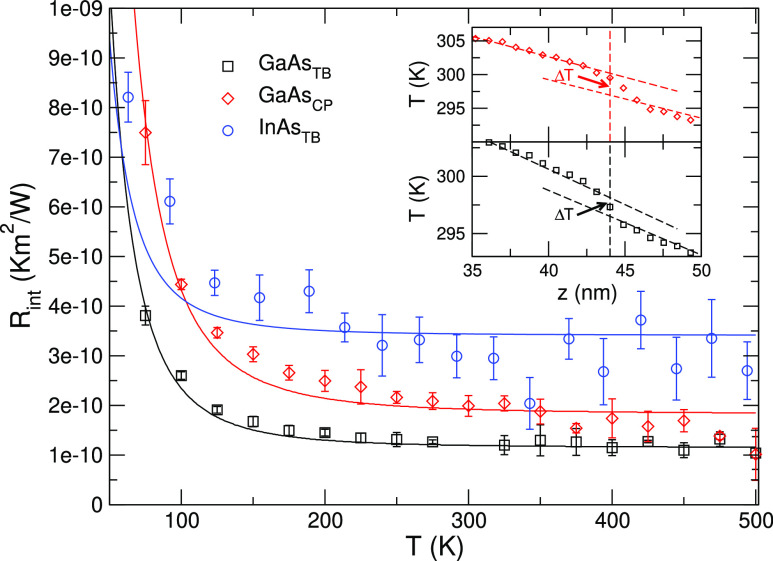
TBR as a function of temperature of a single twin boundary in GaAs and InAs and of a crystal phase interface in GaAs. The continuous lines are fits of the computed data to a *T*^–3^ dependence of the TBR. The inset shows the temperature profile, *T*(*z*), around a crystal phase interface (top) and a twin boundary in GaAs (bottom). The temperature jump, Δ*T*, which is the signature of the TBR and which is used to compute it, is indicated.

As the first step of our study, we have calculated the TBR as a function of the interface temperature of a twin boundary in GaAs and InAs, as displayed in Figure 2. To this end, after reaching the nonequilibrium steady state, we estimate the heat flux from the energy per unit time injected/extracted by the hot/cold thermostats and the thermal gradient from the averaged temperature profile, *T*(*z*); representative examples are provided in the insets. The results obtained are similar to previous reports of GaP and InP, showing that the physical effects are general and do not qualitatively depend on the material or on the classical potential used to describe interatomic interactions (Vashista potential for phosphides,^47,48^ Nordlund for arsenides^42^). In the case of GaAs, we add for comparison a crystal phase interface between segments of zincblende and wurtzite crystals (red diamonds in Figure 2), which is also a class of important interfaces in NW physics.^30,31^ In agreement with the results obtained with GaP and InP, we find a TBR of the order of 2 × 10^–10^ K m^2^ W^–1^, smaller than conventional heterointerfaces but slightly larger than the one obtained for the corresponding twin boundary (black squares in Figure 2). The continuous lines are fits of the calculated data to *T*^–3^, the temperature dependence predicted by the same phenomenological models that fail to account for the TBR^6^ of twin boundaries. While this temperature dependence seems to be reasonable for both the twin boundary and the crystal phase interface in GaAs, in the case of InAs, we obtain a somewhat more abrupt saturation to the high-temperature value, preventing a satisfactory fit to a *T*^–3^ decay, particularly at low temperatures.

As mentioned above and discussed in detail in ref (36), the TBR of twin boundaries derives from the rotation of the phonon polarization vectors. At variance with conventional heterointerfaces, local atomic relaxations play a minor role, as changes in bond lengths are typically of the order of hundredths of Å and concern a very narrow region around the interface, which is not expected to exhibit an appreciable strain.^49^

### Increasing the Number of Twins

Now that we have established that individual twin boundaries in arsenides behave similar to conventional heterointerfaces, though with a smaller associated TBR, we move to the study of multiple interfaces. Our goal is to assess to what extent TSLs, periodic superstructures made of an ordered sequence of twins, behave like conventional superlattices in altering phonon transport. In a first set of computational experiments, we have considered an increasing number of twin boundaries, 1 ≤ *N* ≤ 20, located in the central part of the computational cell. The separation between neighboring twins, δ, is fixed, so that the twinned region has a thickness equal to *N*δ. We take δ = 29.4 Å for GaAs and 31.5 Å for InAs, that is, three unit cells along the [111] crystal axis. Notice that the size of the computational cell along the transport direction has been doubled with respect to the one used for the results of Figure 2. In this way, we guarantee that the twinned region is sufficiently separated from the thermostats, also for the largest values of *N*.

We computed Δ*R* = *R*_*N*_ – *R*_0_, where *R*_*N*_ and *R*_0_ are the thermal resistances of a system with *N* and zero twin boundaries. The thermal resistances are computed as *I*/Δ*T*, where *I* is the heat current and Δ*T* is evaluated between the values of the *z* coordinate axes, *z*_*i*_ and *z*_*f*_, taken to be sufficiently far from the thermal reservoirs to avoid the usual non-linearities of the temperature. We considered two different values of the average temperature *T*_M_ = (*T*_H_ + *T*_C_)/2: a low-temperature case, with *T*_M_ = 100 K, and a higher temperature *T*_M_ = 300 and 250 K, for GaAs and InAs, respectively, which is of the order of the Debye temperature of each material (*T*_D_ = 360 K for GaAs and *T*_D_ = 280 K for InAs).

**Figure 3:**
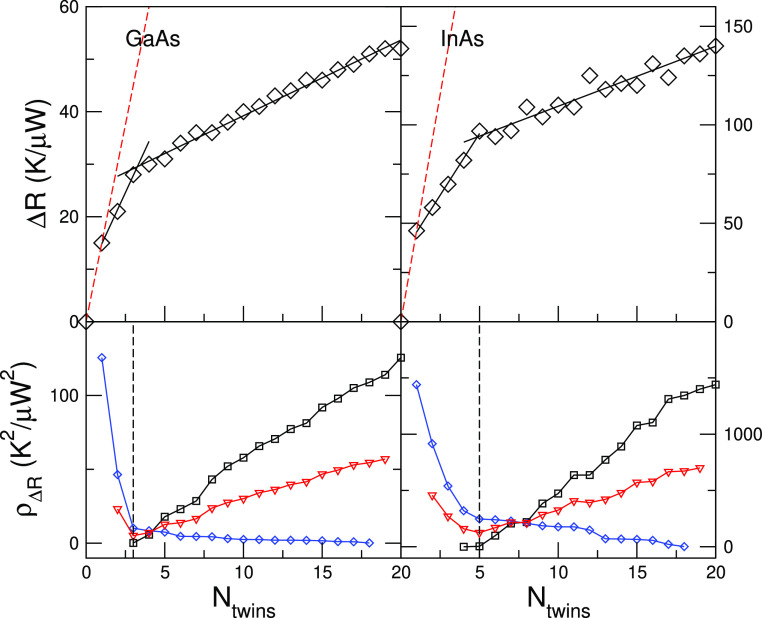
(Top) Thermal resistance as a function of the number of twins in GaAs and InAs, at low temperatures. Δ*R* is defined as *R*_*N*_ – *R*_0_, where *R*_*N*_ and *R*_0_ are the thermal resistances of a system with *N* and zero twin boundaries. (Bottom) Mean of the residual squares of two linear fits of Δ*R*[1, *N*] and Δ*R*[*N*, 20] as a function of *N* (red triangles); the individual residual squares of each fit is also shown (black squares and blue diamonds). The mean has a minimum at *N* = 3 and *N* = 5 for GaAs and InAs, respectively. The corresponding linear fits are shown in the upper panels with a dashed line.

The results for GaAs and InAs at *T*_M_ = 100 K are shown in Figure 3. If each twin boundary acted as an independent scatterer for phonons, Δ*R* should increase linearly and its slope would be related to the TBR of an individual twin. The expected dependence of Δ*R* in this limiting transport regime is indicated with a dashed red line. However, the contribution of the subsequent 2–4 twins to Δ*R* after the first one is quite lower than the contribution of the single twin, for both materials (see Figure 3), an indication of the interaction between neighboring twins for the chosen value of δ. Now, when more than 3–5 twins are stacked together, a collective effect builds up and the slope of Δ*R* is further reduced, indicating yet another change in the transport regime. In order to establish in a quantitative way for which value of *N* the slope changes, we have carried out two linear fits of the computed Δ*R* in the intervals [1, *N*] and [*N*, 20], varying *N*, and plotted the mean residual of squares. We found that the best fits are obtained for *N* = 3 and *N* = 5 for GaAs and InAs, respectively. We also checked if the fit can be improved significantly by assuming more than two linear regressions, but this was not the case. We interpret this behavior in the following manner: when there are up to 3–5 twins, most phonons keep their coherence when traversing the region. With a larger number of twins, a significant fraction of phonons with high contribution to the heat flux start to scatter inside the “metamaterial” region, changing the nature of transport and thus the slope of Δ*R*.

**Figure 4:**
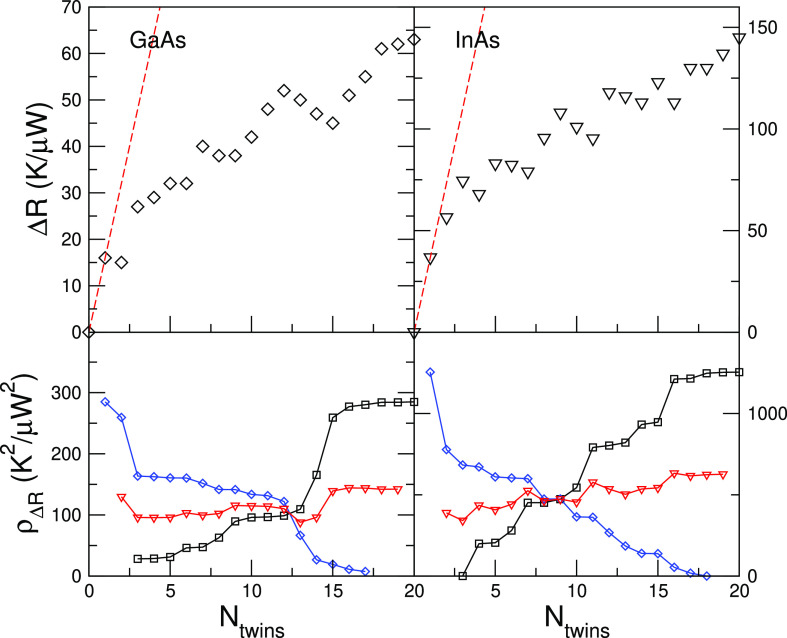
(Top) Thermal resistance as a function of the number of twins in GaAs and InAs, at room temperature. Δ*R* is defined as *R*_*N*_ – *R*_0_, where *R*_*N*_ and *R*_0_ are the thermal resistances of a system with *N* and zero twin boundaries. (Bottom) Mean of the residual squares of two linear fits of Δ*R*[0, *N*] and Δ*R*[*N*, 20] as a function of *N* (red triangles); the individual residual squares of each fit is also shown (black squares and blue diamonds). No clear minima emerge.

In the higher temperature case, where *T*_M_ = 250 or 300 K, such a distinction between two transport regimes is more difficult to make. While by visual inspection it seems that for both materials the slope changes at *N* ∼ 3, no clear minima of the mean of the residual squares emerge (see Figure 4). On the other hand, the oscillations of Δ*R* with *N* are indicative that substantial interference effects persist, and indeed, also in this case, Δ*R* is systematically lower than the independent scatterers regime (red dashed line in Figure 4). Yet, these effects are hardly visible in our lower temperature results, suggesting that further investigation is needed to reveal the nature of these oscillations.

We recall here that at low temperatures, the results obtained from classical molecular dynamics cannot be taken quantitatively. Assuming that atoms move according to Newton’s laws implies that phonon population follows Maxwell–Boltzmann, rather than Bose–Einstein, statistics, and this is a good approximation only at sufficiently high temperatures. Simplified schemes to correct for these effects include a temperature renormalization^50−53^ (the results obtained at a nominal temperature *T*_MD_ are actually valid at a different temperature, *T*_Q_) or using quantum, rather than classical, specific heat, when it is required for the calculation of thermal conductivity.^54,55^ However, even within the more general discussion of Berens et al.,^56^ the result of properly accounting for quantum effects is a temperature-dependent correction of the thermodynamic variables calculated. The results of Figure 3, however, have all been obtained at the same temperature, and thus, any small correction to the computed values would affect in a similar way all data points. Also, it should be kept in mind that we are not concerned here with the specific values of Δ*R* but rather with highlighting different transport regimes. In this sense, our results are reliable and provide a valuable insight into the underlying physics.

### Temperature Profiles

**Figure 5:**
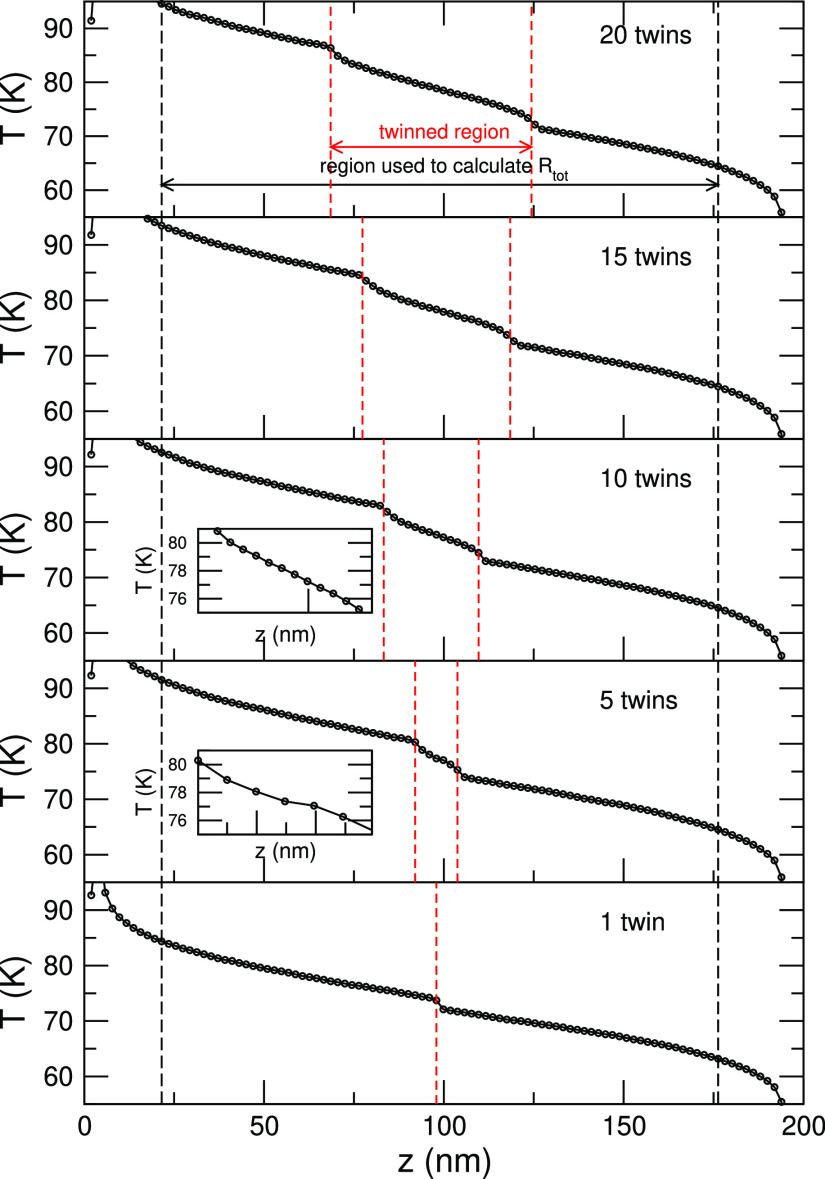
Temperature profile, *T*(*z*), for a GaAs system with *N* = 1, 5, 10, 15, and 20 twins with a constant intertwin separation, δ, and with *T*_M_ = 100 K. The red dashed lines indicate the position of the first and last twin boundaries (i.e., the thickness of the twinned region). The coordinates, *z*_*i*_ and *z*_*f*_, used to evaluate Δ*T* to yield Δ*R* in Figure 3 are marked by the red dashed lines. Inset displays a zoomed view of *T*(*z*) of the twinned region.

The spatial dependence of the temperature along the transport direction is essential in NEMD not only to estimate the TBR but also to compute the thermal conductivity of a homogeneous system. Indeed, even when the temperature of the reservoirs is fixed, one must rely on the thermal gradient of a central region of the simulation cell, rather than on the nominal thermal bias, *T*_H_ – *T*_C_. Additionally, *T*(*z*) conveys an important physical insight in the presence of interfaces. In Figure 5, we plot *T*(*z*) for a GaAs system with 1, 5, 10, 15, and 20 twin boundaries at *T*_M_ = 100 K. The case with one single twin resembles the temperature profile shown in the inset of Figure 2, although here, results have been obtained in a larger computational cell. The temperature discontinuity is small but clearly observable, and it occurs exactly where the twin boundary is located. Next, we consider the case of five twin boundaries. Although it is not possible to observe the signature of the five interfaces, it is clear that within the twinned region, *T*(*z*) still exhibits a certain structure (see the zoomed view in the inset). In the rest of the cases displayed here (*N* > 5), on the other hand, the twinned regions behave at all effects as a metamaterial with its own thermal resistance. Phonons experience a TBR between pure GaAs segments and a central TSL segment. Remarkably, the TBRs to enter/exit the TSL region are, though small, clearly visible and similar in all the cases. Conversely, within the twinned region, *T*(*z*) is linear, as expected in a homogeneous material (see the inset for *N* = 10).

The analysis of Figure 5 helps rationalize the dependence of Δ*R* presented above. Once the twinned region is seen by the propagating phonons as a segment of TSL with its own resistivity, ρ_TSL_, the total resistance is simply
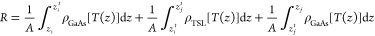
1where *L*_*z*_ = *z*_*f*_ – *z*_*i*_ is the total length probed, *A* is the cross section, and ρ_GaAs_ is the temperature-dependent thermal resistivity of GaAs; the initial and final coordinates of the TSL segment are  and . If, for simplicity, we drop the temperature dependence of ρ_TSL_ and ρ_GaAs_, the resistance simply reads *R* = *N*δρ_TSL_/*A* + (*L*_*z*_ – *N*δ)ρ_GaAs_/*A*. Therefore, if we look back at Figure 3, we can distinguish two transport regimes: at first, Δ*R* increases because the number of twin interfaces increases; next, it increases because the length of the more resistive TSL segment increases, and thus, its relative weight on the total resistance, *R*, is higher. In the first case, phonons see the twin boundaries as interfering scatterers, and the increase of Δ*R* is dictated by the complex interplay of the individual twins; in the second case, the twinned region reaches sufficient thickness to be seen as a TSL metamaterial with its own conductivity, which depends on the superlattice design parameters, for example, the period.

These considerations suggest that a different balance between the TBR of the individual interface and the resistivity of the ideal superlattice, that is, with a very large *N*, could result in a different dependence of Δ*R* on the number of twins. In particular, if the TSL was considerably less resistive than the pure untwinned systems, after the collective interface behavior shows up, it should first hit a maximum and then decrease.

Finally, we observe that plots of *T*(*z*) in Figure 5 indicate that non-linear effects deriving from the use of a finite thermal bias appear to be negligible.

### Increasing the Density of Twins

**Figure 6:**
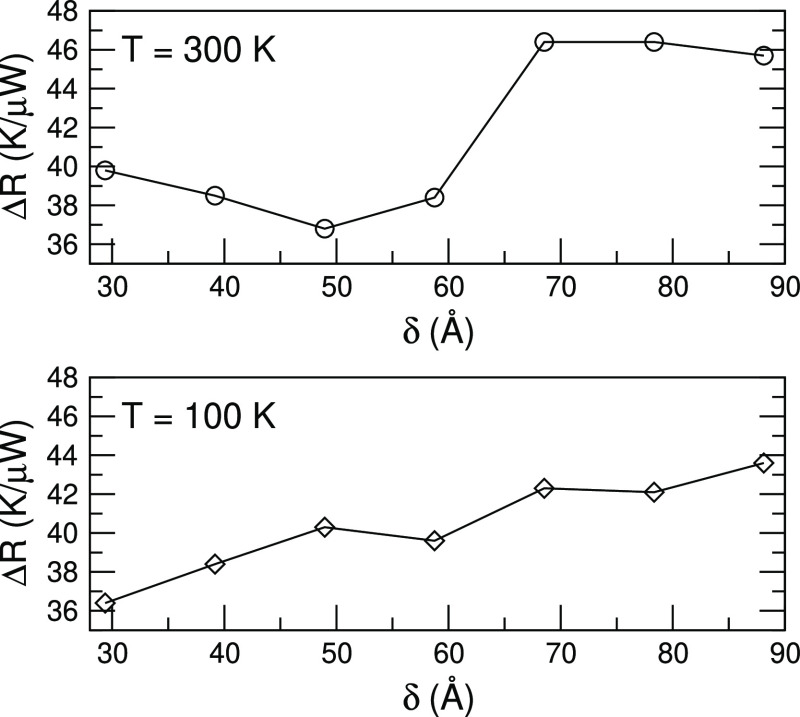
Thermal resistance as a function of the intertwin separation, δ, for a GaAs system with 6 twin boundaries and for (top) *T*_M_ = 300 K and (bottom) 100 K.

Finally, in order to further corroborate our conclusions, we report the results obtained in a different kind of computational experiments. Now, rather than varying the number of twins and fixing their separation, we do the opposite: we consider a fixed number of six twin boundaries and vary gradually the separation between neighboring interfaces. If the transport regime was fully incoherent and each interface scattered phonons independently from the others, the total thermal resistance should be constant, as all the systems contain the same number of twin boundaries. Our results for GaAs are displayed in Figure 6 and capture a rather different situation. While for large separations (δ > 60 Å), the additional resistance introduced by the twin boundaries is almost constant, when they are brought together, it decreases, a clear indication that Δ*R* does not simply result from the sum of independent scattering events. It is interesting that here the trend is quite clear also for a room temperature case, while such an observation was not fully conclusive when we varied the number of twin defects (see Figure 4).

**Figure 7:**
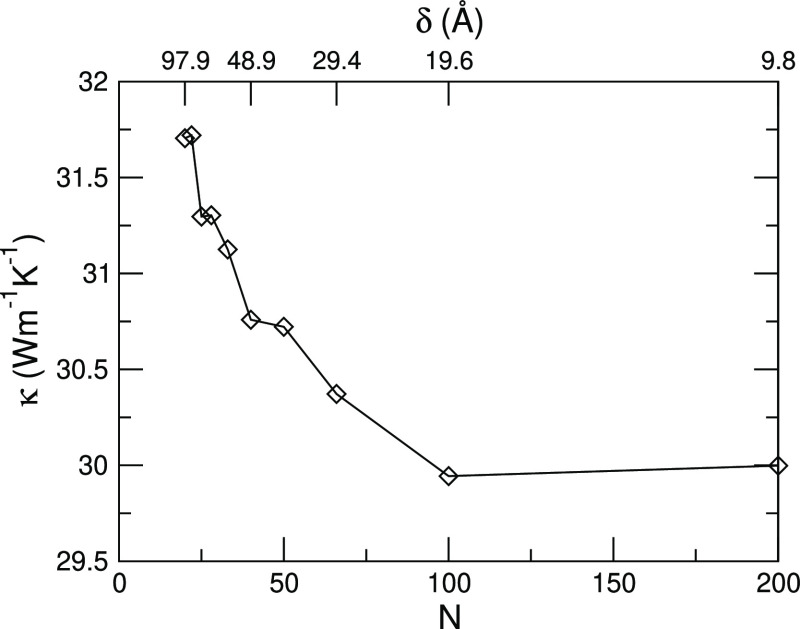
Thermal conductivity of a GaAs TSL at room temperature as a function of the number of twins (lower axis) and the separation between adjacent twins, δ (upper axis). The period of the TSL is *L* = 2δ.

Notice that the twin configurations studied thus far prevented us to compute the thermal conductivity, κ, which is ill-defined in nonhomogeneous materials. Therefore, we performed an additional set of calculations, considering systems where the periodic sequence of twin defects spans the whole NW length, so that the thermal conductivity of the TSL (that now occupies the entire NW) can be defined and calculated. In this case, the TSL morphology and properties are fully defined by the twin periodicity, that is, twice the separation between neighboring twins (see the sketch in Figure 1). As the length is kept constant, to have the same finite-size effects, changing the periodicity results in a different number of twin interfaces. Our results at room temperature are displayed in Figure 7. As it can be seen there, when the period *L* = 2δ decreases, the thermal conductivity decreases, because the number of twins increases. However, this initial abrupt decrease at first slows down (at *N* ∼ 50) and then saturates. Indeed, when doubling the overall number of interfaces in short period TSLs (*N* going from 100 to 200), κ does not change (as a matter of fact, it even slightly increases, from 29.9 to 30 W m^–1^ K^–1^).

It is interesting to compare these results with those recently reported by Ghukasyan and LaPierre.^57^ They studied fully twinned GaAs TSL, like those we considered in Figure 7, though they explicitly accounted for the NW geometry, studying systems with diameters of at most 10 nm. They observe a deep and well-defined minimum at small periods, while we see a saturation. This suggests that the sawtooth faceting that naturally arises in this kind of nanostructures^32^ plays an important role in the observation of this behavior and thus in highlighting the incoherent-to-coherent transport crossover. This observation agrees with the results of Xiong et al.,^58^ who studied Si NW TSL and reported a minimum in the thermal conductivity only when the axial zigzag structure was considered (indeed, they rationalize their results on the basis of geometric arguments). Notice that Ghukasyan and LaPierre report a minimum at a period of approximately 50 Å, while in our calculations, κ saturates at δ = 19.6 Å, which corresponds to a period *L* ∼ 40 Å (Figure 7). This agreement indeed indicates that the underlying physics is the same in both cases, though we expect our results to be a better approximation for large diameter NWs, similar to those that are routinely grown experimentally.^27,59−61^

## Conclusions

In summary, we have carried out computational experiments based on nonequilibrium molecular dynamics and provided clear fingerprints of the fact that TSLs behave similarly to their conventional counterparts, where segments of different materials repeat periodically. Namely, we have shown that (i) isolated twin defects have a small but finite TBR; (ii) when a sufficiently high number of twin defects are stacked close to each other, phonons see the twinned region as a homogeneous (meta)material; (iii) the way a given number of twin defects scatter phonons depends on their density, indicating the so-called particle-to-wave crossover. These observations corroborate recent experimental reports^35^ and indicate that crystal-phase engineering can become an effective way to design materials with desired phononic properties and to manipulate phonons similar to conventional superlattices, but with additional advantages, such as defect-free and atomically abrupt interfaces.
